# Twelfth Annual Commencement of the Medical Department of the University of Buffalo

**Published:** 1857-03

**Authors:** 


*Twelfth Annual Commencement of the Medical Department of the University of Buffalo.*—The commencement exercises took place on Wednesday evening, February 25th.

On Monday the candidates underwent a severe examination before the Faculty. On Wednesday morning another examination took place before the Faculty and Curators, and fifteen candidates were recommended to the Council for the Degree of Doctor of Medicine. The Council met in the afternoon, and confirmed the recommendation. The conferring of the degrees took place at Kremlin Hall, in the evening. The platform was occupied by the Chancellor of the University, Hon. Millard Fillmore, with members of the Council, the Faculty, and the Board of Curators.

A large and fashionable audience filled the hall. The Rev. Dr. Burtis made the opening prayer, after which Mr. Fillmore, in his capacity of Chancellor, read an address to the graduates, replete with sound advice, and full of appreciation of the responsible duties they were about to assume. He then formally conferred the degrees on each of the following candidates, in succession:

Charles S. Boynton, Elizabethtown, Ind.
George W. Ewell, Central Square, N. Y.
George H. Calkins, Waupeca, Wis.
David W. Hotaling, Waterport, N. Y.
Peregrine M. Mann, St. Thomas, C. W.
William Q. Mansfield, Buffalo.
Henry Nichell, Buffalo.
Job E. Ostrander, St. Thomas, C. W.
Sylvester Rankin, Buffalo.
Albert B. Robinson, Ware, Mass.
James S. Rundell, Watertown, N. Y.
William B. Sprague, Pavillion, N. Y.
William B. Thomas, Rush, N. Y.
John B. Tweedale, Washington, C. W.
John S. Welles, Castile, N. Y.

Prof. Flint then delivered the charge to the graduates. Premising that they had doubtless looked sufficiently upon the sunny side of medical practice, he proceeded to point out to them many of the difficulties and disappointments which they must be prepared for. He gave a rehearsal of the misconceptions and the lack of appreciation, which are the lot of the most fortunate and successful physicians. Speaking from a large experience, he portrayed the heart-aches which embitter the life of medical men — holding the attention of his audience by occasional irony or apt illustration. Professor Flint's style was remarkably good. Without a shadow of attempt at eloquence, his diction was neat and chaste, and his manner of delivery impressive. All were delighted with both manner and matter. Of the latter we can only say that we hope it may be remembered and acted upon.

The Rev. Orlando F. Starkie closed the evening's exercises with a benediction, and the young doctors were sent forth with sober faces.

